# Photosynthesis and Fine Root Growth Dynamics of Soybean in Walnut-Soybean Agroforestry System

**DOI:** 10.1155/2022/2246824

**Published:** 2022-02-10

**Authors:** Bin Liu, Pengxiang Gao, Shuoxin Zhang

**Affiliations:** College of Forestry, Northwest A&F University, Yangling, Shaanxi 712100, China

## Abstract

Agroforestry system is regarded as a promising practice in sustainable agricultural management. However, the effects of long-term tree-based intercropping on crop remain poorly understood, especially in the Loess Plateau (China). In this study, the impacts of photosynthetic and respiration rate were determined by the portable photosynthesis system (Li-6400), and the effects of the root growth dynamics of soybean in the walnut-soybean intercropping system were measured by soil auger and WinRHIZO root analysis system, in the Loess Plateau. The results showed that soybean reached the highest net photosynthetic rate during flowering period, with the net photosynthetic rate of intercropped soybean, which was 20.40 *μ*mol·m^−2^·s^−1^, significantly higher than that of its monocropped counterpart. Soybean biomass reached the maximum during the pod-bearing period, with intercropped soybean biomass being 25.49 g, significantly higher than that of its monocropped counterpart. The mean diameter and increased density of soybean fine roots reduced along with increased soil depth. Both the diameter (0.43 mm) and increased density (930 cm/dm3) of intercropped soybean fine roots were evidently higher than those of monocropped soybean (0.35 mm, 780 cm/dm3). With increasing cropping years, fine roots of intercropped soybean tended to be mainly distributed in soil at a depth between 0 and 20 cm from the fifth year. Collectively, compared with soybean monoculture, walnut-soybean agroforestry system is more conducive to soybean growth in the Loess Plateau.

## 1. Background

The Loess Plateau is the cradle of the Chinese nation and the ancient civilization [1]. Competition and reciprocity among species determine the stability and sustainability of the system itself, in a multispecies ecosystem [2]. As an artificial multispecies vegetation system, the agroforestry system entails different functional organs in the spatial niche, varied functional activity cycles on the temporal dimension, competition adaptability in term of biomorphic function, and species distribution patterns on the spatial dimension [3, 4]. Theoretically, these give rise to a composite system. The competition and interplay between different species in a multispecies ecosystem determines the stability and sustainability of the system itself [5, 6]. Considering the potential benefits of tree-based intercropping systems, an appropriate agroforestry system can be a foundation for effective resource utilization. However, in actual applications, the benefits of agroforestry may disappear as tree shades can reduce crop photosynthesis and competition between root systems affects crop's absorption of water and nutrients.

This paper takes the walnut-soybean agroforestry system in the Loess Plateau of China as the research object and discusses the influence of soybean photosynthesis and the competitive relationship between soybean and walnut roots. Maximize crop yields with actionable recommendations for smart urban agroforestry systems and smart cities.

## 2. Methods

### 2.1. Plant Materials and Site Description

Focusing on the walnut-soybean agroforestry system in the Loess Plateau of China, the competitive relationship between soybean and walnut root system was studied (11 years) in the walnut-soybean agroforestry system; four experimental plots (50 × 50 m) were established in 2007. In order to guide farmers in the Loess Plateau region to conduct reasonable agroforestry management and considering the canopy density of walnut forest, 1–4 years after walnut planting is a reasonable number of years for agroforestry, and explore the photosynthesis and biomass of soybean by building 4 experimental plots (50 × 50 m) in 2016.

The research area is located in the southern part of the Loess Plateau, belonging to the temperate continental monsoon climate. Before the establishment of the experimental plots in 2007 and 2016, the experimental fields had been rotations for 10 years in winter wheat and corn. The soil physical and chemical properties, soil organic matter content, soil water content, altitude, and slope direction were basically the same. The experimental areas of the study was located in Zhangjiagou village (34°19 ′36 ″N, 107°37′ 16″ E) and Liujiagou village (34°19 ′41 ″N, 107°38′ 40″ E) in Qishan county, Shaanxi Province. The annual average sunshine hours are 2185 h, the average temperature is 11.6°C, the extreme maximum temperature is 41.4°C, the extreme minimum temperature is −20.6°C, and the frost-free period is 198 days. The mean annual precipitation is 679 mm, which can basically meet the needs of crop growth. However, due to the influence of monsoon climate, the annual seasonal distribution is not uniform. The precipitation in June to September accounts for about 70% of the annual precipitation. In the test area, the soil was mainly made of loess mother material, with organic matter content of 2.45%, total nitrogen content of 0.116%, and total phosphorus content of 0.098% [7].

Soybean is the major crop in the researched area. The crop under tree canopies selected in this research was soybean (Qindou 8). We have selected the most widely grown local walnut variety in this region of Xiangling. The walnut trees were planted in 2016 (1a) at 3 m × 6 m row spacing. The width of soybean alleys was 4.8 m, while that of walnut tree strips was 1.2 m. Soybean was planted on June 6, 2017, at 30 cm ^*∗*^ 30 cm row spacing. The planting density of monocropped soybean was the same as that of its intercropped counterpart.

### 2.2. Photosynthetic and Respiration Rate Sampling

On three consecutive sunny days, the net photosynthesis rate (Pn), stomatal conductance (Gs), respiratory rate (Tr), and intercellular CO_2_ concentration on the second fully expanded leaf of soybean, which were selected during 9 : 00–11 : 00 a.m., were measured with the portable photosynthesis system (Li-6400; LI-COR Inc., Lincoln, NE, USA).

### 2.3. Biomass Sampling

The sampling was collected when there were about four compound leaves (branching), inflorescence began to appear (flowering) and all inflorescence dropped (podding) of the soybean. The biomass was measured when the sample weight is dried (80°C) to a constant weight.

### 2.4. Soil Sampling

Soil moisture content of 20 sampling points distributed in an s-shaped form was established in the sample plot, where the soil auger was used to obtain samples at the depth of 0–20, 20–40, and 40–60 cm. One hundred grams of soil was weighed and placed in the oven at 105 ± 2°C to dry the constant weight (*W*). Samples were taken during the branching, flowering, and pod-bearing periods, respectively:

(1) $$\begin{array}{c} \text{soil moisture content}=\frac{100-W}{W}\times 100\%, \end{array}$$

where *W* means the constant weight.

### 2.5. Root Sampling

**Figure 1:**
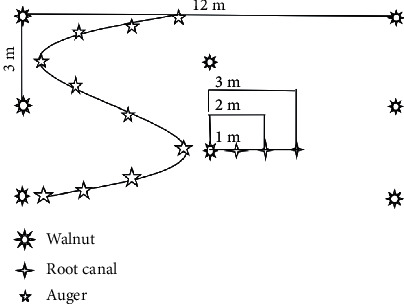
Schematic diagram of sampling points.

Root samples were taken with a soil auger. The total sampling depth was 60 cm, with 10 cm for each layer. The sampling points of “s” soil auger were reset for each sampling (Figure 1), far away from fringe areas, and avoided the last time the sample point. Samples were taken during the branching, flowering, and pod-bearing periods, respectively.

Soybean fine roots were prepared by soaking, washing, and separating procedures, and on this basis, fine roots with a diameter of less than 2 mm were selected. WinRHIZO root analysis system was applied to obtain the morphological indicators of fine roots, such as root length density and fine root diameter.

After the fixed sample plots were established, a minirhizotron system (BTC-100 Borescope Root Ecology Monitoring System) was employed to carry out in situ observation of seasonal growth dynamics of roots. Root imaging data were collected every 10 days over an entire growth cycle. Root length parameter was obtained using the Winrhizo-tron-MF to calculate root length density, which was followed by a calculation of fine root length for the observation period.

Pianka [8] niche overlap formula (symmetric *α* method) is adopted to calculate the competition intensity index among walnut-soybeans, and the formula is shown as follows:

(2) $$\begin{array}{c} A_{ih}=\frac{\sum_{j=1}^{r}P_{ij}P_{hj}}{\sqrt{\sum_{j=1}^{r}P_{ij}^{2}\sum_{j=1}^{r}P_{hj}^{2}}}. \end{array}$$

*A*_*ih*_ = *A*_*hi*_, and *A*_*ih*_ ≤1, *r* is depth of soil layer, and *r* ≤ 3, *P*_*ij*_ and *P*_*hj*_ are, respectively, the proportion of species *i* (length density of soybean) or species *h* (length density of soybean) using the *j* (length density of soybean and walnut) resource bit in its utilization of all resource bits. The Pianka method is insensitive to the number of individuals of a population or its quantitative characteristics of a population in a community, but it can objectively reflect the extent of niche overlap between populations and the changes of niche overlap relation between populations and is used to measure the degree of competition of Shared resources among populations under the condition of resource shortage.

The data of the test were analyzed by SAS 8.2 software for variance analysis, and the figures were made by OriginPro9.0 software. Set the soil layers in question as 4 soil layers of 0–20, 20–40, and 40–60 cm based on data analysis results.

## 3. Results

The most evident difference between intercropped and monocropped soybean lies in a larger competitiveness of intercropped soybean in terms of transpiration rate, net photosynthesis rate, and stomatal conductance during the flowering period (Table 1). For both intercropped and monocropped soybean, the transpiration rate, net photosynthesis rate, and stomatal conductance increased and then decreased over time, reaching their maximum values during the flowering period.

The dry weight of intercropping soybean was significantly heavier than that of monoculture soybean at the podding stage (Table 2). At flowering stage, the dry weight of under ground of intercropping soybean was significantly higher than that of monoculture soybean. For intercropping soybean and monoculture soybean, the fresh weight, dry weight of single plant, and dry weight of above ground and under ground of soybean increased with time and reached the maximum at the podding stage of soybean.

The competition intensity between walnut and soybean decreased with the increase of soil depth (Table 3). The competition between walnut and soybean roots is the most intense at 0–20 cm.

**Figure 2:**
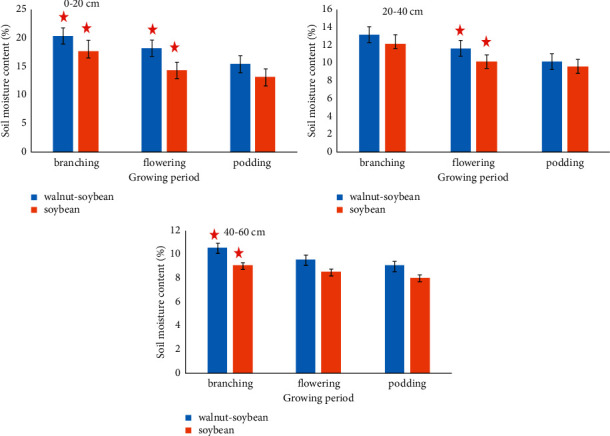
The relative soil moisture content in different soil layers. The same asterisk indicates a significant influence, *P* < 0.05.

The soil moisture content gradually decreases with the increase of soybean growth period and soil depth (Figure 2). At 0–20 cm, the moisture content of the walnut-soybean agroforestry system was significantly higher than that of the single-cropping soil at the flowering stage (*P* < 0.05).

**Figure 3:**
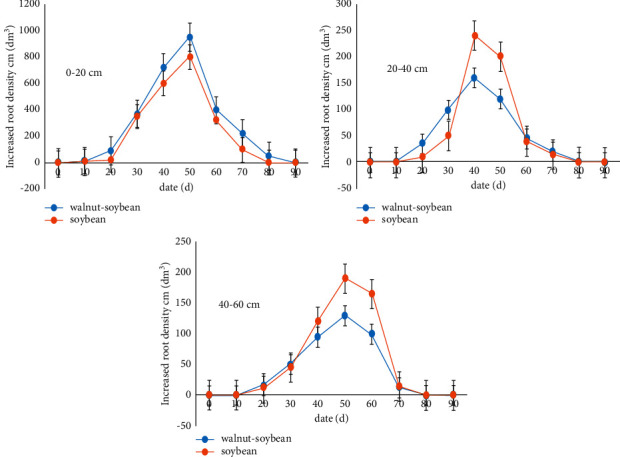
The relative density of increased root of soybean in different soil layers.

Both intercropped and monocropped soybean had only one root growth peak at the soil depth of 0–60 cm, with the peaks being reached basically at the same time (Figure 3). Starting from the 5th year, intercropped soybean had a significantly greater root growth peak (*P* < 0.05) at 0–20 cm soil depth than its monocropped counterpart. Starting from the 50^th^ day after soybean planting, the monocropped soybean had a significantly greater root growth peak (*P* < 0.05) at 20–60 cm soil depth than its intercropped counterpart.

**Figure 4:**
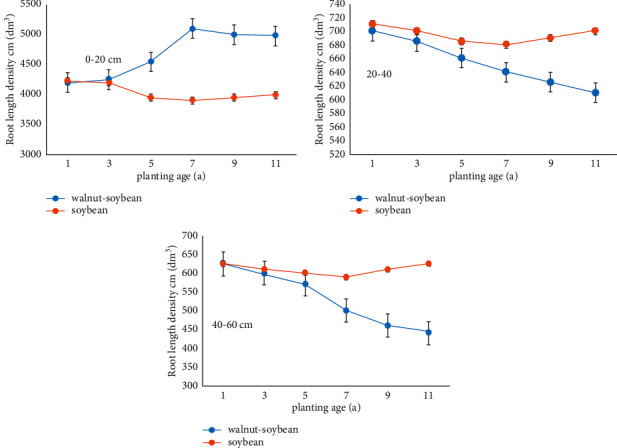
The relative density of soybean root in different soil layers.

There were no significant changes in soybean fine root length densities at 0–60 cm soil depth with the increasing planting years (Figure 4). The root length density of intercropped soybean at 0–20 cm soil depth increased with the increasing planting years. Starting from the 5th year, the root length density of intercropped soybean was significantly higher than its monocropped counterpart (*P* < 0.05). The root length density of intercropped soybean at 20–60 cm soil depth decreased with the increasing cultivation years and started to be significantly lower than that of the monocropped soybean (*P* < 0.05) from the 7th year.

**Figure 5:**
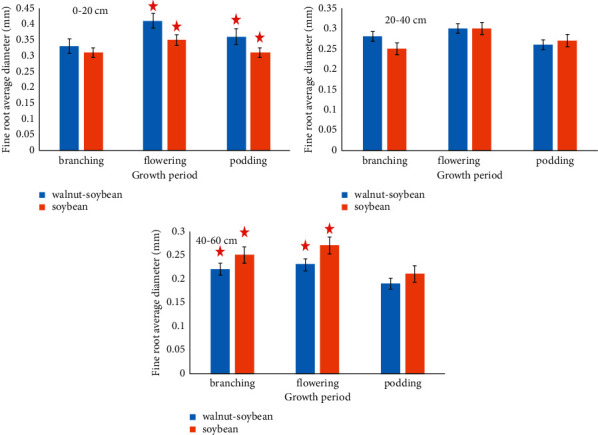
The relative average diameter of fine root of the soybean in different soil layers. The same asterisk indicates a significant influence, *P* < 0.05.

The mean fine root diameter of intercropped soybean at 0–20 cm soil depth was significantly larger than that of the control group (Figure 5). The mean fine root diameter of intercropped soybean during the flowering period was significantly larger than that of the control group (*P* < 0.05), but the same diameter at 20–40 cm soil depth was smaller than that of the monocropped soybean during the sowing period. The mean fine root diameter of intercropped soybean at 40 cm soil depth was significantly smaller than that of the control group (*P* < 0.05).

## 4. Discussion

According to the preliminary data published by the General Administration of Customs of China in 2016, soybean imports have exhibited a growing trend over recent years, and soybean is proven to generate more economic benefits than other crops [9, 10]. To alleviate poverty and soybean import pressure, the Chinese government has started to encourage soybean cultivation among farmers. Exploring the interspecies competitive pressure exerted on soybean in the walnut-soybean agroforestry system is of great significance.

Shading affects the net photosynthesis rate of crops, which indirectly affects crop yields. Namirembe [11] found that the walnut-soybean agroforestry system had a limited shading influence on soybean, which is consistent with our experimental results. A study conducted by Miller and pallardy [12] indicated that competition over water resources represented a key factor influencing crop yields in the walnut-soybean agroforestry system. An appropriate level of shading effectively prevents soil moisture evaporation, increases soil moisture concentration, contributes to the increase in net photosynthesis rate, and thus improves the biomass of soybean [13]. Such a conclusion is also supported by the results of this research. According to the study of Zhang et al. [14], yields of intercropped soybean decreased by 29% compared with those of monocropped soybean. This may be attributable to the relatively thinner soil layer in Zhang 's experiment site located in the Taihang Mountain region, which can result in unfixed ecological niche of soybean roots and thus higher pressure of interroot competition. Results showed that the walnut-soybean agroforestry system can improve soybean yields effectively.

The growth and root length density peaks of intercropped soybean at 0–20 cm soil depth were higher than those of its monocropped counterpart, and the growth and fine root length density peak values of intercropped soybean at 20–60 cm soil depth were lower than its monocropped counterpart, which are consistent with findings of Zhang et al. [15] and Wang et al. [16]. Yang et al. [17] argued that root growth was associated with soil moisture, water obtained by soybean from the soil would be predominantly used for root growth, and insufficient soil moisture would stimulate root growth. Interspecies competition between the root systems of a walnut-soybean agroforestry system is quite intense at 0–20 cm soil depth, which stimulates, to some extent, the growth of soybean roots. Guan et al. [18] and Li et al. [2] asserted that the walnut-soybean agroforestry system effectively increased the moisture content of the 20–60 cm soil layer, and consequently, the root length density of monocropped soybean would be higher than that of the intercropped soybean at this soil depth. As such, the walnut-soybean agroforestry system can effectively improve the root length density of soybean and increase the crop's absorption and utilization of water from soil.

In a walnut-soybean agroforestry system, soybean root systems could achieve rapid growth within a short period of time, thus gaining an advantage in the interspecies competition. Results from this study showed that, with the increasing planting years in a walnut-soybean agroforestry system, the central distribution of roots exhibits a declining trend for walnut and a climbing trend for soybean. The results of this research are consistent with those of Fan et al. [19], Ma et al. [20], and Gao et al. [21]. To sum up, due to the interspecies competition between the root systems in a walnut-soybean agroforestry system, the roots of intercropped soybean tend to be distributed in the 0–20 cm soil layer. In the 5th operating year of the walnut-soybean agroforestry system, a unique ecological niche of soybean roots starts to take shape in the soil.

Results from this study showed that the mean diameter of soybean fine roots reaches its maximum value during the flowering period, which may be due to the fact that the flowering season falls in late July when rainfall in the Qishan region reaches its maximum intensity, in the 0–60 cm soil layer. Abundant soil moisture reduces the pressure of interspecies competition between soybean roots [22], explaining why the mean diameter of soybean fine roots reaches its maximum value during the flowering period. In the 0–60 cm soil layer, the mean diameters of both intercropped and monocropped soybean reduce along with the increasing soil depth, which may be due to decreased water and nutrients with the increase in soil depth [15]. In the 0–20 cm soil layer, the mean root diameter of intercropped soybean significantly increases compared with that of its monocropped counterpart. This may be caused by the phenomenon that the remnant pore spaces of walnut roots amid their turnover processes are utilized by soybean roots, and a relatively loose soil environment contributes to the increase of mean diameter of soybean fine roots. These are consistent with findings of Xun et al. [23] and Wang et al. [24]. In the 40–60 cm soil layer, the mean root diameter of intercropped soybean is significantly lower than that of its monocropped counterpart. Soybean fine roots are mostly distributed in the 0–20 cm soil layer [25]. However, a study conducted by Wang et al. [26] explored the mean fine root diameter of wheat in a walnut-wheat agroforestry system, finding that, in the 0–60 cm soil layer, the mean fine root diameter of intercropped wheat is larger than that of its monocropped counterpart. This may be because that the walnut-soybean agroforestry system cannot give rise to a stable ecological niche of roots as the walnut-wheat agroforestry system does. Therefore, the smaller mean fine root diameter of intercropped soybean in the 40–60 cm soil layer is a product of a survival strategy adopted by soybean roots in order to cope with the fierce interspecies competition [27–29]. In short, the walnut-soybean agroforestry system—whose operation originated from late July when interspecies competition was the lowest—can provide favorable soil conditions for roots of intercropped soybean, which further contributes to soybean roots' general water and nutrient absorption from soil in the 0–60 cm soil layer.

## 5. Conclusions

In conclusion, we argue that, with the increasing planting years, a fixed ecological niche for soybean roots will be established in the walnut-soybean agroforestry system. The interspecies competition of soybean roots in the walnut-soybean agroforestry system is favorable, to some extent, for root growth, which further facilitates soybean roots in the intercropping system to absorb water from soil, thereby improving soybean yields.

## Figures and Tables

**Table 1:** The photosynthetic factors of soybean.

Measurement		Transpiration rate (m mol·m^−2^·s^−1^)	Net photosynthesis rate (*μ*mol·m^−2^·s^−1^)	Stomatal conductance (mol m^−2^·s^−1^)	Intercellular CO2 concentration(**μ**l L^−1^)
Branching	CK	1.43 a	12.94 a	155.18 a	106.94 a
	T	1.29 ab	14.94 a	172.24 a	93.31 a

Flowering	CK	4.29 a	16.93 a	187.40 a	195.44 a
	T	3.43 b	20.40 b	212.36 b	188.14 ab

Podding	CK	2.92 a	15.63 a	178.26 a	263.72 a
	T	2.29 ab	16.31 ab	189.31 a	213.24 b

The data in the table are the mean value of different treatments. The letter after data expression Duncan's multiple comparison results. Those data in the same column with the same letter indicate that there is no significant difference (*P* > 0.05). T denotes walnut-soybean; CK denotes soybean.

**Table 2:** The biomass of soybean.

Measurement		Fresh weight of single plant (g)	Dry weight of above ground (g)	Dry weight of under ground (g)	Dry weight of single plant (g)
Branching	CK	10.43 a	2.18 a	0.70 a	2.83 a
	T	10.49 a	2.20 a	0.71 a	2.94 a

Flowering	CK	26.16 a	4.73 a	1.17 a	5.90 a
	T	29.72 ab	5.02 ab	1.67 b	6.68ab

Podding	CK	65.09a	16.14 a	5.24a	21.39a
	T	74.76 ab	18.65 b	6.83 b	25.49 b

The data in the table are the mean value of different treatments. The letter after data expresses Duncan's multiple comparison results. Those data in the same column with the same letter indicate that there is no significant difference (*P* > 0.05). T denotes walnut-soybean; CK denotes soybean.

**Table 3:** The competition between the root of walnut and soybean in agroforestry system.

Soil depth	0–20 cm	20–40 cm	40–60 cm
Root density walnut	75.632 ± 0.515	40.735 ± 0.273	17.256 ± 0.209
Soybean (cm/dm^3^)	4317.235 ± 2.749	694.317 ± 1.416	601.403 ± 1.037
Competition intensity index	0.637 ± 0.026A	0.386 ± 0.033B	0.215 ± 0.013C

The data in the table are the mean value of different treatments. The letter after data expresses Duncan's multiple comparison results. Those data in the same column with a different letter indicate that there is a significant difference (*P* < 0.05).

## Data Availability

No analytical permission has been obtained from the data provider because of trade confidentiality.

## References

[1] Zhang J.-T. (2010). The relationship between environmental decline and the destruction of vegetation in Loess Plateau. *Journal of Shanxi University(Natural Science Edition)*.

[2] Li Q., Dong B., Qiao Y., Liu M., Zhang J. (2010). Root growth, available soil water, and water-use efficiency of winter wheat under different irrigation regimes applied at different growth stages in North China. *Agricultural Water Management*.

[3] Ong C. K., Corlett J. E., Singh R. P., Black C. R. (1991). Above and below ground interactions in agroforestry systems. *Forest Ecology and Management*.

[4] Chen G. P., Chai Q., Niu J. Y. (2007). Research on root temporal and spacial distribution of intercropped cereal and legume crops. *Acta Agriculturae Boreali-occidentalis Sinica.*.

[5] Bhagwat S. A., Willis K. J. (2008). RSPO principles and criteria for sustainable palm oil production. *Conservation Biology*.

[6] Tsonkova P., Böhm C., Quinkenstein A., Freese D. (2012). Ecological benefits provided by alley cropping systems for production of woody biomass in the temperate region: a review. *Agroforestry Systems*.

[7] Iuss Working Group Wrb (2015). World reference base for soil resources 2014, update 2015. international soil classification system for naming soils and creating legends for soil maps.

[8] Pianka E. R. (1973). The structure of lizard communities. *Annual Review of Ecology and Systematics*.

[9] Peng X., Zhang Y., Cai J., Jiang Z., Zhang S. (2009). Photosynthesis, growth and yield of soybean and maize in a tree-based agroforestry intercropping system on the Loess Plateau. *Agroforestry Systems*.

[10] Department of foreign trade and economic statistics (2017). *China Foreign Trade Statistics Yearbook 2016*.

[11] Namirembe S. (1999). Tree management and resource utilization in agroforestry systems with Senna spectabilis in the drylands of Kenya.

[12] Miller A. W., Pallardy S. G. (2001). Resource competition across the crop–tree interface in a maize-silver maple temperate alley cropping stand in Missouri. *Agroforestry Systems*.

[13] Luo W.-X. (1995). *Study on Construction Model of Eco-Economical Type protection forest System on WeiBei of Loess Plateau*.

[14] Zhang J. S., Meng P., Xin X. B., Yin C. J. (2011). Effects of appleginger inter-cropping in the hilly land of Taihang Mountain. *Scientia Silvae Sinica*.

[15] Zhang J. S., Meng P., Yin C. J., Ma X. Q., Feng W. D. (2002). Spatial and temporal distribution characteristics of wheat roots in apple-wheat intercropping. *Forest Research*.

[16] Wang W., Chen W.-C., Wang K.-R., Xie X.-l., Yin C.-M., Chen A.-l. (2011). Effects of long-term fertilization on the distribution of carbon, nitrogen and phosphorus in water-stable Aggregates in paddy soil. *Agricultural Sciences in China*.

[17] Yang S. Y., Yan P., Mei X. Y., Sun X. B. (2007). Effects of different soil water deficits on roots of winter wheat. *Journal of Triticeae Crops*.

[18] Guan X. J., Zhao S. W., Wang J. Z., Li B. C. (2001). Effects of water deficits on roots and crown of winter wheat during its different development stages. *Acta agriculture boreali-sinica*.

[19] Fan W., Lu Q., Gao X. R. (1999). Distribution pattern and growing dynamics of the roots system in apple-wheat intercropping system. *Acta Ecologica Sinica*.

[20] Ma C. M., Zhuo M. P., Liu C. P. (2009). Root distribution characteristics of *Juglans regia* in monoculture and intercropping. *Journal of Beijing Forestry University*.

[21] Gao L., Xu H., Bi H. (2013). Intercropping competition between apple trees and crops in agroforestry systems on the Loess Plateau of China. *PLoS One*.

[22] Wang C., Huang L., Wang Z. Y., Qiao X. J. (2007). Monitoring and analysis of electrical signals in water‐stressed plants. *New Zealand Journal of Agricultural Research*.

[23] Xun H. S., Gao L. B., Bi H. X., Yun L., Bao B. (2012). Fine root distribution and underground competition in walnut-soybean intercropping system. *Chinese Journal of Ecology*.

[24] Wang B. J., Zhang W., Ahanbieke P. (2014). Interspecific interactions alter root length density, root diameter and specific root length in jujube/wheat agroforestry systems. *Agroforestry Systems*.

[25] Xun H. S., Bi H. X., Gao L. B. (2013). Distribution and morphological variation of fine root in a walnut-soybean intercropping system in the Loess Plateau of China. *International Journal of Agriculture and Biology*.

[26] Wang L., Gao P. X., Zhong C. G. (2018). Growth dynamics and competitive strategies of fine roots in a walnut-wheat agroforestry system. *Acta Ecologica Sinica*.

[27] Sun G. Y., Zhang R. H., Huang Z. W. (2002). Soybean root distributions in meadow-blackland and albic-soil. *Chinese Journal of Oil Crop Sciences*.

[28] Myers D. B. (2005). Soybean root distribution in claypan soils.

[29] Yan C. J., Wang W. B., Tu X. J. (2013). Effect of drought stress at different growth stage on yield and root characteristics of soybean. *Soybean science*.

